# Multiples épulis

**DOI:** 10.11604/pamj.2016.25.15.10626

**Published:** 2016-09-20

**Authors:** Hakima Elmahi, Fatima Zahra Mernissi

**Affiliations:** 1 Service de Dermatologie, CHU Hassan II, Fès, Maroc

**Keywords:** Epulis, gencive, tumeur bénigne, Epulis, gum, benign tumour

## Image en médecine

L’épulis (épi = dessus, oulon=gencive) est une pseudotumeur bénigne hyperplasique circonscrite des gencives, la plus fréquente, elle répondra à deux critères qui font l’unanimité: un critère topographique: la localisation de l’épulis au niveau du collet d’une ou de deux dents contiguës; un critère de bénignité bien précisé par LAUFER: « l’épulis est en effet une tumeur qui ne récidive pas après exérèse complète, qui ne donne pas de métastases, ni d’envahissement ganglionnaire ». Classiquement, le rôle de l’inflammation seul est retenu pour expliquer l’étiopathogenie. Mais de troubles humoraux souvent observaient a type de variation ou perturbation endocrine, hypovitaminose C, troubles hématologiques. L’épulis s’observe à tout âge et se présente cliniquement sous forme de masse charnue rouge foncé très vascularisée, saignant facilement au contact, circonscrite sessile ou pédiculée, Histologiquement, l'épulis distingue différentes formes: simples, inflammatoires, vasculaires ou angiomateuses, fibreuses, à cellules géantes ou épulis à myéloplaxes. Son traitement est l'exérèse chirurgicale. Nous rapportons le cas d'une patiente de 76 ans, sans antécédent particulier, consultait pour des tumeurs de la gencive évoluant depuis 3 ans. L'examen clinique objectivait au niveau de la gencive inférieure et inferieure deux masses indolores, exophitiques, en forme de framboise, ferme, à base sessile saignant au contact, avec un mauvais état bucco-dentaire. Le reste de l'examen clinique était normal, une radiographie panoramique réalisée était sans particularité. Le diagnostic d'épulis a été retenu. Ainsi l'exérèse chirurgical avec examen anatomopathologique a confirmé le diagnostic. L’évolution était bonne avec des soins dentaires réguliers.

**Figure 1:**
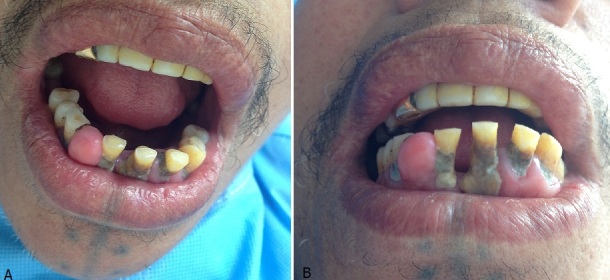
A) tumeur nodulaire de la gencive inferieure; B) multiples nodules de la gencive inferieure

